# Variance reduction in randomised trials by inverse probability weighting using the propensity score

**DOI:** 10.1002/sim.5991

**Published:** 2013-09-30

**Authors:** Elizabeth J Williamson, Andrew Forbes, Ian R White

**Affiliations:** a Department of Epidemiology and Preventive Medicine, Monash UniversityMelbourne, Victoria, Australia; b Melbourne School of Population and Global Health, University of MelbourneVictoria, Australia; c MRC Biostatistics UnitCambridge, U.K.

**Keywords:** variance estimation, baseline adjustment

## Abstract

In individually randomised controlled trials, adjustment for baseline characteristics is often undertaken to increase precision of the treatment effect estimate. This is usually performed using covariate adjustment in outcome regression models. An alternative method of adjustment is to use inverse probability-of-treatment weighting (IPTW), on the basis of estimated propensity scores. We calculate the large-sample marginal variance of IPTW estimators of the mean difference for continuous outcomes, and risk difference, risk ratio or odds ratio for binary outcomes. We show that IPTW adjustment always increases the precision of the treatment effect estimate. For continuous outcomes, we demonstrate that the IPTW estimator has the same large-sample marginal variance as the standard analysis of covariance estimator. However, ignoring the estimation of the propensity score in the calculation of the variance leads to the erroneous conclusion that the IPTW treatment effect estimator has the same variance as an unadjusted estimator; thus, it is important to use a variance estimator that correctly takes into account the estimation of the propensity score. The IPTW approach has particular advantages when estimating risk differences or risk ratios. In this case, non-convergence of covariate-adjusted outcome regression models frequently occurs. Such problems can be circumvented by using the IPTW adjustment approach. © 2013 The authors. Statistics in Medicine published by John Wiley & Sons, Ltd.

## 1 Introduction

Propensity scores were introduced in 1983 as a tool to estimate the causal effect of a binary exposure or treatment from non-randomised data 1. In recent years, the use of propensity scores in the analysis of non-randomised studies has increased dramatically. The propensity score is the probability of receiving the treatment conditional on measured characteristics, a probability that can be estimated from the data by modelling the treatment allocation as a function of measured characteristics. Rosenbaum and Rubin demonstrated the important property that at any value of the propensity score, the distributions of confounders included in the model are balanced between treatment groups, a property that carries over to the estimated propensity score as long as the propensity score model is correctly specified 1. This property allows unbiased estimation of the causal treatment effect at each value of the propensity score. This leads to various ways of using the propensity score to estimate the treatment effect, including matching or stratifying on the estimated propensity score or inverse probability weighting by functions of the estimated propensity score 2,3.

In contrast to the traditional approach of building a statistical model for the outcome variable conditional on measured confounders, therefore, the propensity score approach instead models the treatment allocation process. In a simple individually randomised controlled trial, the treatment allocation process is known. Further, because treatment is randomised, there is no confounding. Thus, it is unclear how the propensity score approach could be usefully applied to the analysis of individually randomised controlled trials. In this context, Senn 4 ,Section 7.2.13 had described propensity scores as ‘superfluous and misleading’.

In this paper, we argue that propensity scores are a useful tool for the analysis of individually randomised controlled trials. This does, however, require a change in perspective. Rather than viewing the propensity score as a method of bias reduction as for non-randomised studies, we will view the propensity score analysis as a method of covariate adjustment aimed at increasing precision of the treatment effect estimate. In order to achieve this, we will move away from the philosophy of modelling the treatment allocation process and towards the idea of modelling chance imbalance or designed balance (such as stratified randomisation) of prognostic variables between treatment groups. In this context, the utility of propensity scores in randomised trials becomes much clearer. Although adjustment for baseline characteristics is unnecessary to avoid bias in randomised studies, it can greatly enhance the precision of the treatment effect estimate. Currently, such adjustments are often performed in individually randomised controlled trials using linear regression models for continuous outcomes or logistic regression for binary outcomes 5. We will call these approaches ‘covariate-adjustment’ in order to distinguish them from the propensity score estimators described in the succeeding text.

Our aim is, therefore, to suggest that a propensity score approach, specifically inverse probability of treatment weighting (IPTW), is an attractive way of implementing pre-specified adjustment for baseline characteristics or factors used to stratify randomisation in individually randomised controlled trials. However, we demonstrate that particular care needs to be taken with the method of variance estimation in order to capitalise on the benefits of IPTW. We organise the paper as follows. In Section 2, we define the inverse probability of treatment weighted estimator. In Section 3, we calculate the large-sample marginal variance for this propensity score estimator. In Section 4, we apply our variance results to show that for continuous outcomes, the propensity score estimator has similar statistical properties to the covariate-adjusted (linear regression) estimator, but that in order to achieve comparable precision to the covariate-adjusted estimator, the standard error must be correctly estimated; naive estimators of the standard error can greatly understate the precision of the propensity score estimator. In Section 5, we provide some guidelines for how propensity scores should be used in randomised trials, and how this differs from their standard use in observational studies. We present results from a small simulation study in Section 6. In Section 7, we demonstrate these methods using an individually randomised trial of physiotherapy for the treatment of adhesive capsulitis (a painful shoulder condition). We end, in Section 8, with a discussion.

## 2 A propensity score (inverse probability of treatment weighting) estimator of treatment effect

### 2.1 Notation

We consider a trial comprising *n* participants who are individually randomised to two treatment arms. For participant *i*, the binary treatment allocation is *Z*_*i*_ (0=placebo, 1=active), the outcome is *Y*_*i*_ and we have a vector of measured baseline characteristics that we wish to adjust for, **x**_*i*_ = (*X*_0*i*_,*X*_1*i*_,..,*X*_*pi*_)^ ⊤ ^. In the regression models in the succeeding text, we set *X*_0*i*_ = 1 for each participant in order to include an intercept term in each regression model that involves the vector **x**_*i*_. Each participant has two possible or *potential* outcomes: the one that would occur under allocation to placebo and the other that would occur under allocation to the active treatment. We will denote these two potential outcomes by *Y*_0*i*_ and *Y*_1*i*_, respectively. For each participant, we can observe only one of these potential outcomes because only one treatment is allocated. We can quantify the causal treatment effect by contrasts of 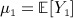 and 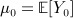. For continuous outcomes, the mean difference *δ*_1_ = *μ*_1_ − *μ*_0_ is often of interest. For binary outcomes, *δ*_1_ is the risk difference. The marginal risk ratio or odds ratio are also frequently used to quantify the treatment effect. We will define these two estimands on the natural logarithm scale because this is the scale on which inference will later be performed. We will define the log marginal risk ratio and log marginal odds ratio as *δ*_2_ = *log*(*μ*_1_ / *μ*_0_) and *δ*_3_ = *log* ({*μ*_1_ / (1 − *μ*_1_)} / {*μ*_0_ / (1 − *μ*_0_)}), respectively.

### 2.2 Assumptions

Before estimating a causal treatment effect, we must make several assumptions. The first two assumptions, which in a non-randomised setting must be made but cannot be verified, are automatically satisfied by a randomised design. Firstly, we make the consistency assumption, which states that the observed outcome *Y*_*i*_ is equal to the appropriate potential outcome: if *Z*_*i*_ = *z* then *Y*_*i*_ = *Y*_*z*_. This is guaranteed by design in a randomised trial, because the treatment is well-defined and under the control of the investigators 6. The positivity assumption states that each participant must have a non-zero probability of receiving either treatment allocation, which again is guaranteed by the design of the trial 7. We further assume that the data (*Y*_*i*_,*Z*_*i*_,**x**_*i*_) are independently distributed for different subjects indexed by *i* and that the treatment received by one participant does not affect the potential outcomes of another participant, where the latter is often termed as the stable unit treatment value assumption (SUTVA). We note that SUTVA includes the consistency assumption. The SUTVA assumption is often made in both non-randomised and randomised settings. The final assumption, that of strongly ignorable treatment assignment, states that there is (in expectation) no unmeasured confounding: (*Y*_0*i*_,*Y*_1*i*_) ∐ *Z*_*i*_ | **v**_*i*_, where **v**_*i*_ is a (vector) subset of the measured baseline characteristics **x**_*i*_, with ∐ indicating conditional independence. This assumption cannot be verified in non-randomised settings but is satisfied by randomisation of treatment with **v**_*i*_ being the empty set 8.

### 2.3 An inverse probability of treatment weighted estimator

We will define the propensity score as the conditional probability of receiving the active treatment given the measured baseline characteristics, *e*(**x**) = *Pr*(*Z* = 1 | **x**). Suppose that the binary treatment indicator *Z* follows a logistic model parametrized by ***α*** = (*α*_0_,*α*_1_, ⋯ ,*α*_*p*_)^ ⊤ ^, so that *log*{*e*(**x**) / (1 − *e*(**x**))} = **x**^ ⊤ ^***α***. We can obtain maximum likelihood estimates 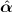 by fitting a standard logistic regression model. We will estimate the propensity score for participant *i* as 

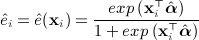

Although we have chosen to use a logistic regression model for the propensity score, other approaches are possible. Various authors have considered the application of different methods including neural networks, recursive partitioning and boosting 7, 9–11 and they have identified some situations in which these methods can perform better than logistic regression. In the current paper, however, we consider only the logistic regression model.

Once the propensity score has been estimated, the IPTW estimators for the two marginal means are 

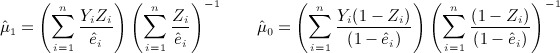
2
We use appropriate contrasts of these estimators to obtain estimators for the treatment effect, 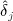, for *j* = 1,2,3. These will be referred to as the IPTW or propensity score, treatment effect estimators.

We note that the IPTW estimator is derived from the equality 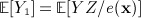. To see this, by conditioning on **x**, we have 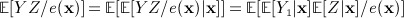 where the last equality follows from the consistency and strongly ignorable treatment assignment assumptions. Because 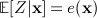, the right-hand side cancels to give 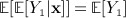, from which we have 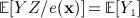 as desired. Similarly, 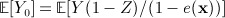.

We can also obtain the estimates given in Equation (1) by fitting a linear or binomial regression model, as appropriate, for outcome *Y* on treatment allocation *Z* with no other independent variables, applying the following probability weights: 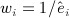 if *Z*_*i*_ = 1, and 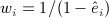 if *Z*_*i*_ = 0, using the appropriate link function (identity for *δ*_1_, log-link for *δ*_2_ and logit link for *δ*_3_). However, we note that the sandwich variances typically produced by statistical software for such models are incorrect because they do not allow for the estimation of the propensity score; they are typically conservative, sometimes greatly so. We will return to this point in the next section.

One attractive feature of the IPTW approach is that a treatment effect estimate can be obtained even when the outcome is a rare binary outcome, and we wish to adjust for many covariates. Equation (1) is simply two weighted means, which will be defined provided that neither treatment arm is empty, and estimation of the propensity score does not involve the outcome. Thus, the convergence problems, which can occur when fitting outcome regression models to rare binary outcomes, will not occur for IPTW estimators derived from Equation (1).

If the propensity score model includes only a constant term, the estimated propensity score will be a constant, in which case the estimators in Equation (1) become the unweighted sample means, and the treatment effect is estimated by the standard unadjusted difference in means, risk difference, risk ratio or odds ratio. We will call these unadjusted estimators 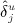, for *j* = 1,2,3. In the succeeding discussion, we compare the large sample marginal variances of the unadjusted and propensity-score adjusted (IPTW) treatment effect estimators, 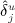 and 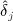, respectively.

## 3 Variance estimation for inverse probability-of-treatment weighted estimators

Using the theory of M-estimation 12, Lunceford and Davidian 13 have calculated the large-sample marginal variance of the difference in means for continuous outcomes. This variance can also be applied to estimates of the risk difference for binary outcomes. We now extend this calculation to risk ratios and odds ratios.

### 3.1 Marginal large-sample variance of inverse probability-of-treatment weighted estimates

Substituting estimated propensity scores, 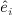, derived from a logistic regression model as described earlier, into Equation (1) to estimate *μ*_1_ and *μ*_0_ is equivalent to simultaneously solving the estimating equations 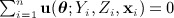 for the parameter ***θ*** = (*μ*_1_,*μ*_0_,***α***^ ⊤ ^)^ ⊤ ^, where 

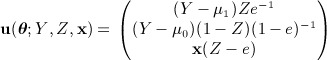

The first two components of **u** are scalars, and the third is a *p* × 1 column vector. The resulting estimator 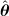 is asymptotically normally distributed, with large-sample variance equal to *n*^ − 1^**A**^ − 1^**BA**^ − ⊤ ^ where 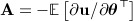 and 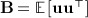, with the derivative evaluated at the true value of the parameter ***θ*** and the expectations taken over the true distribution of the data. 12 These matrices are calculated in Appendix 1, and standard matrix multiplication is applied to give the marginal covariance matrix for 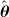. From this, we can obtain 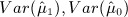 and 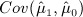. Because the treatment effect estimate 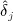 is simply a function of 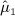 and 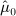, we then apply the delta method to obtain the large-sample marginal variances of 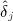, for *j* = 1,2,3. The Appendix shows that this gives 

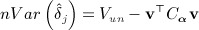
4
where 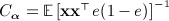 is the large-sample covariance matrix of the estimated propensity score parameters 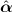, and 

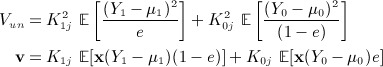

with 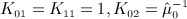 and 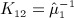, and 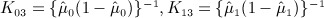. We note that these constants are simply the derivatives of the link functions that would be used to estimate these parameters via a generalised linear model, evaluated at the expected mean under universal treatment (*μ*_1_) or no treatment (*μ*_0_), respectively.

Repeating the aforementioned variance calculation including only a constant in the propensity score model shows that the standard unadjusted estimates 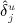 have large-sample variance equal to 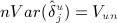. Because the second term of (2) is the negative of a quadratic form around a positive definite matrix, 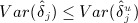. Thus, using the propensity score to adjust for baseline characteristics results in an estimated treatment effect (mean difference, risk difference, risk ratio or odds ratio) at least as precise as the unadjusted treatment effect estimate.

We note that this result—that adjustment will not result in an increase in the variance of the treatment effect estimate—does not hold in non-randomised settings. When treatment is not randomised, adjusting for a baseline characteristic that predicts treatment allocation but not outcome can result in an increase in variance 14. In a simple randomised trial, the true propensity score *e* is not a function of any baseline characteristics; thus, the first term in the aforementioned Equation (2), *V*_*un*_, remains the same no matter what variables are included in the propensity score model. The second term of Equation (2) is always negative; thus, adjustment can never increase the variance. In no-randomised settings, the true propensity score is a function of the characteristics included in the propensity score. In this case, when predictors of treatment but not outcome are included, the first term is typically increased, whereas the magnitude of the second term is unchanged; thus, the variance is increased. The effect of adjustment on the variance of the treatment effect estimate in non-randomised settings is discussed in detail elsewhere 15.

### 3.2 Sample estimate of the marginal large-sample variance of inverse probability-of-treatment weighted estimates

Variance formula (2) could be used to estimate the variance of the IPTW estimator in a sample of data, by replacing unknown quantities with sample estimates. However, this requires the estimation of the two potential outcomes, *Y*_1_ and *Y*_0_, which requires further assumptions to be made. For example, when estimating the mean difference, we could assume a constant treatment effect giving 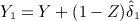. An alternative approach, which avoids the estimation of these potential outcomes, is to repeat the aforementioned calculation replacing the expectations by sample averages and leaving expressions in terms of observable quantities (i.e. *Y* and *Z*, not *Y*_1_ and *Y*_0_). 12 This estimates the variance of 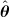 by 

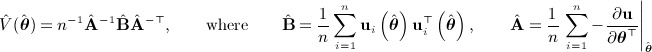
6
As previously discussed, we can then apply the delta method to obtain the following sample estimate of the variance of the treatment effect estimator 

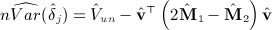
7
where, letting 
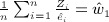
 and 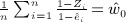, we have 

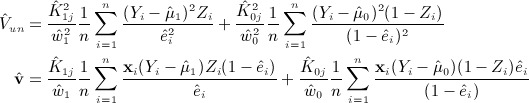

and 

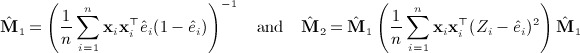

We obtain the 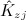
′s by replacing *μ*_0_ and *μ*_1_ by their sample estimates. For continuous outcomes, setting 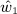 and 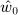 to their expectation of 1 in the formula for 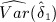 gives the variance estimate proposed by Lunceford and Davidian 13, which, in practice, gives very similar variance estimates to our formula.

### 3.3 Consequences of ignoring the estimation of the propensity score

We now suppose that the treatment effect estimate was obtained via IPTW estimates of the two means, *μ*_1_ and *μ*_0_, but that the estimated propensity scores were replaced by specified propensity score values, 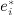. Repeating the aforementioned calculation, removing the component of the estimating equation that estimates the propensity score parameters shows that our new treatment effect estimator, 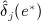 say, would have an estimated variance of 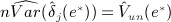, where 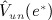 is obtained by replacing 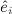 by 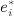 in 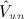. In particular, this shows that if the propensity score is estimated but we treat the estimated scores, 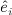 as known quantities the sample estimate of the variance would be 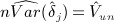. This is a sample estimate of the variance of the unadjusted estimator. Therefore, by treating the estimated propensity scores as known quantities, we would appear to lose all the precision that has been gained by the IPTW adjustment for baseline characteristics.

Although this result may sound counterintuitive—that ignoring a source of variation will artificially decrease the precision—we note that the estimation of the propensity score is not a source of variation in the usual sense. In a simple randomised trial, the true propensity score is 0.5 for all participants; thus, using this in inverse weights will result in unadjusted estimators of treatment effect. Inverse weighting using the true propensity score does not, therefore, take account of chance imbalance of prognostic baseline characteristics. The variability in the estimated propensity score simply reflects chance imbalances in the prognostic baseline characteristics that are included in the propensity score model across treatment groups. The increased balance of these covariates caused by the weighting using these estimated propensity scores (in comparison with using the true propensity score, i.e. calculating unadjusted estimates) results in increased precision of the treatment effect estimate. Failure to account for the estimation of the propensity score will not take into account the decrease in precision obtained by the re-balancing of prognostic characteristics created by weighting using the estimated (rather than true) propensity score; thus, the precision will be falsely decreased.

If we instead estimate the IPTW treatment effect by fitting a probability-weighted outcome regression model as described previously, the same considerations apply. Standard robust or empirical sandwich variance estimators for the estimated regression parameters typically treat the probability weights—which in this case are functions of the estimated propensity scores—as known values. To obtain the correct variance for the estimated regression parameters from these probability-weighted models, the full sandwich variance estimator based on all the estimating equations, including the components estimating the propensity score, involved in the estimation process should be used. This is our sample variance estimator given in Equation (4). We note that the recently released version 13.1 of statistical software package Stata 16 has added the IPTW estimators of treatment effect with the correct variance as part of the new ‘teffects ipw’ command.

Ignoring the estimation of the propensity score will have the greatest consequences with a continuous outcome, where adjustment produces the largest reduction in variance. Using the naive variance estimator, 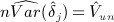, will result in very high coverages for confidence intervals and conservative *p*-values. Comparisons between methods, based on this incorrect variance estimator, will erroneously lead to the conclusion that IPTW adjustment offers no increase in precision compared with a simple unadjusted comparison between treatment groups.

## 4 Comparing the variance of the inverse probability-of-treatment weighted estimator with covariate-adjusted estimators

### 4.1 Continuous outcomes

We now consider a scenario where a linear regression model holds for the outcome and the treatment allocation probability does not depend on baseline characteristics, that is, *e*(**x**) = *e*, a constant, as with simple randomisation. We calculate the large-sample marginal variances of the IPTW and covariate-adjusted estimators in this setting and show that they are equal.

We let *X* denote a characteristic measured at baseline that we wish to adjust for. This will often be the measurement of the outcome variable taken at baseline. We set **w** = (1,*Z*,*X*)^ ⊤ ^ and assume that the following mean model is correct: 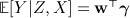, where ***γ*** = (*γ*_0_,*γ*_*Z*_,*γ*_*X*_)^ ⊤ ^. We also assume that the variance of *Y* does not depend on *X* or *Z*, in which case 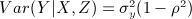, where 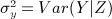 and *ρ* = *Corr*(*Y*,*X* | *Z*) [17, Section 10.3]. We denote the marginal mean and variance of *X* by *μ*_*x*_ and 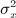, respectively.

#### 4.1.1 Covariate-adjusted estimator

Kenward *et al*. 18 found that the marginal variance of the covariate-adjusted estimate of treatment effect 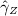, from a linear regression model of *Y* on *Z* and *X*, conditional on the number of participants in the active arm 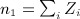 and the placebo arm 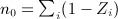 is given by 

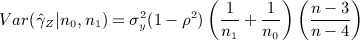

(Note that we have replaced the authors’ original notation of *σ*_*TT*.0_ by the equivalent in our notation 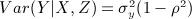). To obtain the unconditional large-sample variance, we appeal to the equality 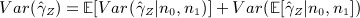. The second term of this equation is zero, because 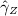 is unbiased. For the first term, as *n* → ∞ , *n*_1_ / *n* → ^*p*^*e*, and *n*_0_ / *n* → ^*p*^(1 − *e*). By Slutsky′s lemma (*n*_1_ / *n*)^ − 1^ + (*n*_0_ / *n*)^ − 1^ → ^*p*^*e*^ − 1^ + (1 − *e*)^ − 1^. Further, (*n* − 3) / (*n* − 4) → ^*p*^1. So 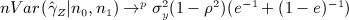. Thus, for large samples, 

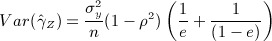
11
We obtain the large-sample marginal variance of the unadjusted (*t*-test) estimator, 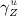, in the same way: 

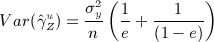
12
We note that these variance results can also be arrived at by applying the M-estimation procedure to the score functions for the linear regression model.

#### 4.1.2 Inverse probability-of-treatment weighted estimator

For the IPTW estimator, 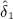, we have 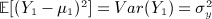. Similarly, 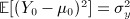. Then 

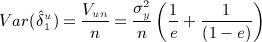
13
For the covariance matrix for the estimated propensity score parameters, 

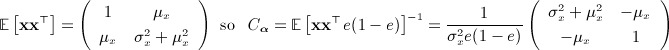


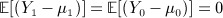
, and 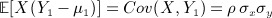, and similarly, 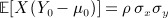. Then **v** = *ρ* *σ*_*x*_*σ*_*y*_(0,1)^ ⊤ ^. Standard matrix multiplication gives 

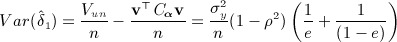
15

#### 4.1.3 Comparison of inverse probability-of-treatment weighting and covariate-adjusted estimators

As expected, the large-sample marginal variances of the two unadjusted estimates (7) and (6) are equal. Perhaps less expected is the fact that the large-sample marginal variances of the covariate-adjusted (5) and IPTW (8) treatment effect estimates are identical. By modelling a known treatment allocation process, we achieve a reduction in variance that is the same as the reduction achieved by standard covariate-adjustment via outcome linear regression modelling, in comparison with the variance of the unadjusted estimate.

### 4.2 Binary outcomes

With a continuous outcome, adjustment does not change the underlying estimand; the marginal and conditional (on characteristics adjusted for) population mean differences are equal. Similarly, when we estimate the risk difference or risk ratio for binary outcomes, the marginal and conditional estimands are equal. However, the marginal and conditional population odds ratios are not equal because of the non-collapsibility of the odds ratio. Typically, the conditional odds ratio is further from the null value of one 19. Therefore, comparisons between variances of marginal and conditional odds ratio estimators are further complicated by the fact that the two estimators are not estimating the same number.

When the treatment probability, *e*, is constant, 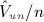 is equal to the usual variances of the unadjusted risk difference, log risk ratio or log odds ratio. We have shown that adjustment for baseline characteristics via the ITPW approach reduces the variance of each of these estimators.

When estimating the covariate-adjusted risk difference or risk ratio, it is unclear what effect adjustment has on the variance of the treatment effect estimator. When estimating the odds ratio via a logistic regression model for the outcome, Robinson and Jewell 20 showed that covariate adjustment increases, or leaves the same, the variance of the estimated log odds ratio for treatment.

The comparison between the variance of the IPTW and covariate-adjusted estimates of the risk difference and risk ratio is therefore unclear. In the case of the odds ratio, adjustment for baseline characteristics decreases the variance of the IPTW estimator but increases the variance for the covariate-adjusted (logistic regression) estimator. Therefore, after adjustment for baseline characteristics, the IPTW log odds ratio is more precisely estimated than the logistic regression log odds ratio. Which estimator has greater statistical power, however, is not clear, because although adjustment increases the variance for the logistic regression estimator, moving from a marginal to a conditional estimator takes the estimand further from the null value, which has the net result of increasing the statistical power to detect the treatment effect 20. However, in this case, the IPTW estimator has the advantage of increasing statistical power without changing the underlying estimand.

## 5 Variable selection for the propensity score model

In this section, we begin by reviewing standard approaches to modelling the propensity score in non-randomised settings. We then propose a slightly modified strategy for individually randomised trials.

In non-randomised settings, to obtain a consistent estimate of treatment effect, the variables included in the propensity score model must satisfy the assumption of strong ignorability of treatment assignment (no unmeasured confounders), which means that all confounders must be included in the model. Often, but not always 21, variables that are predictive only of treatment, or only of outcome, can additionally be included without violating the strong ignorability assumption. Modelling strategies for the propensity score in non-randomised studies generally fall into two categories. In the first, advocated by Rubin 22, only predictors of treatment are included in the propensity score model, allowing the propensity score model to be estimated without reference to the outcome variable. The second, more recent, approach advises the inclusion of predictors of outcome whether or not they are predictive of treatment 23. This latter approach focuses on minimising the variance of the treatment effect estimate: including predictors of outcome but not treatment typically decreases the variance of the estimate of treatment effect while including predictors of treatment but not outcome will generally increase the variance of the estimate of treatment effect 14. Once the candidate variables have been selected for the propensity score model, an iterative procedure is often used to choose the final model. The ‘correct’ propensity score model is the one that achieves balance in the confounders across treatment groups, with the degree of imbalance often assessed using percentage standardised differences 24. If balance is not achieved by the current model, variables can be added or removed, and non-linear terms and interactions can be added or removed until balance is achieved.

In randomised trials, randomisation ensures that the assumption of strong ignorability of treatment assignment is met with no variables included in the propensity score model. The first propensity score modelling approach, including only predictors of treatment, therefore, is not helpful in this context because randomisation ensures that there are none. The second approach, however, is more useful. Prognostic variables for the outcome can be additionally included in the propensity score model without introducing bias, in order to reduce the variance. In particular, variables used to stratify randomisation are typically strongly prognostic. The first approach to propensity score modelling would suggest that because, by definition, stratifying variables are not predictive of treatment allocation, they should not be included in the propensity score model 4. However, taking the second approach, we would advise the opposite: that stratifying variables must be included in the propensity score model. Although inclusion of such variables will not change the point estimate, the variance of the treatment effect estimate will then take account of the fact that this strongly prognostic variable is balanced by design.

Because the randomisation ensures that all imbalance is due to chance, there is no defined correct propensity score model, removing the need for an iterative procedure to identify the correct model. Thus, the propensity score model can, and should, be pre-specified in the same way as a covariate adjustment analysis.

In summary, we suggest the propensity score model should include variables stratified during randomisation and a pre-specified selection of a small number of key prognostic variables measured at baseline. This parallels the advice given for the choice of covariates to include in a covariate-adjusted analysis 25. The propensity score model can thus be completely pre-specified in accordance with standard procedure for randomised trials 26,25. We note that this allows the adjustment (the calculation of the inverse probability weights) to be undertaken prior to the collection of outcome data, thus increasing the amount of pre-specification, in comparison with more traditional methods of adjustment, and avoiding the possibility of the outcome data influencing the statistical models used.

## 6 Simulation study

In this section, we present results from a small simulation study that we undertook to compare the statistical properties of covariate-adjusted estimators with the IPTW estimators.

### 6.1 Description of the simulation study

We randomly allocated treatment, *Z*, by drawing from a Bernouilli (0.5) distribution. We independently simulated four baseline characteristics, *X*_1_,*X*_2_,*X*_3_ and *X*_4_, from a normal distribution with mean 0 and variance of 9. We simulated a continuous outcome variable, *Y*_*cts*_, from a normal distribution with variance of 0.25 and mean given by 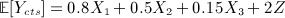. These distributions result in (marginal) correlations within each randomised arm between the outcome *Y*_*cts*_ and the four baseline characteristics *X*_1_,*X*_2_,*X*_3_,*X*_4_ of approximately 0.8,0.5,0.15 and 0, respectively. The true treatment effect, as measured by the mean difference, is 2.

We have drawn a binary outcome, *Y*_*bin*_, from a Bernouilli (*p*_*y*_) distribution, with *p*_*y*_ = (1 + *exp*(1.5 − 0.05*X*_1_ − 0.025*X*_2_ − 0.025*X*_3_ − 0.4*Z*))^ − 1^, resulting in a prevalence of *Y*_*bin*_ of approximately 20%. The true odds ratio, conditional on the three baseline characteristics, for randomised treatment is *exp*(0.4) ≈ 1.49. We approximated the marginal odds ratio from a large simulated dataset, giving *exp*(0.398) ≈ 1.489. So in this scenario, the marginal and conditional odds ratios are almost identical. The true risk ratio and risk difference, also approximated from a large simulated dataset, are *exp*(0.31) ≈ 1.36 and 0.007, respectively.

We have drawn sample sizes of 100, 200 and 1000, giving samples of approximately 50, 100 and 500 per arm. For each sample size, we have drawn 5000 simulated datasets.

We calculated unadjusted estimates of each treatment effect (mean difference, risk difference, risk ratio and odds ratio), then estimates adjusting only for the most prognostic baseline characteristic, *X*_1_, followed by estimates adjusting for all three prognostic baseline characteristics (*X*_1_,*X*_2_ and *X*_3_), and finally estimates adjusting only for a baseline characteristic unrelated to the outcome, *X*_4_. We performed adjustment via covariate adjustment (linear regression or binomial regression with the appropriate link function) and using the IPTW estimators described in this paper.

We used standard variance estimators for the treatment effect estimates obtained from an outcome regression model. For the IPTW estimates, we used the variance formula derived previously (Equation (4)). We calculated the uncorrected robust variance estimate (Equation (4) omitting the second term of the variance formula) for the purposes of comparison. Finally, we calculated the variance from Equation (2), substituting estimates for *μ*_0_,*μ*_1_,*e* and replacing *Y*_1_,*Y*_0_ by estimates (adding (if *Z* = 0) or subtracting (if *Z* = 1) the estimated treatment effect to the linear predictor and, for binary outcomes, drawing a binary outcome from the appropriate Bernouilli distribution). We refer to this as the ‘plug-in’ variance estimator. We used each variance estimator to generate estimated 95% confidence intervals for each simulated sample, on the basis of approximate asymptotic normality except in the case of the linear regression model where the exact *t*-distribution was used, from which estimated 95% coverage probabilities were derived.

### 6.2 Results from the simulation study

For the continuous outcome, estimating the mean difference, there was no bias in any of the estimators at any sample size (Table 1). In general, the incorrect variance estimate for the IPTW treatment effect estimate greatly overestimated the variance, resulting in 95% coverage probabilities close to 1. This was the case in all situations other than when only *X*_4_, a covariate unrelated to the outcome, was adjusted for. In this case, the propensity score adjustment results in improved balance of this non-prognostic characteristic across treatment groups, which has no effect on the precision of the treatment effect estimate. Thus ignoring the effect of the estimation of the propensity score does not result in an incorrect variance in this case. When three baseline characteristics were simultaneously adjusted for, the IPTW approach fractionally over-estimated the variance for a sample size of approximately 50 per treatment arm, resulting in a coverage of 0.97. Adjusting for an unrelated covariate only resulted in a slightly increased variance for smaller sample sizes (empirical variance of 0.347 versus 0.327 for *n* = 50), but no difference for larger sample sizes.

**Table 1:** Estimated mean differences from *n* = 5000 simulated datasets, with sample sizes per arm (*n*) of 50, 100 and 500. The mean treatment effect estimate (Est), the empirical variance across simulations (Emp Var), the mean of the variance estimates (Est Var), and the coverage of the 95% confidence interval calculated from that variance estimate are shown.

Adjustment Method	*n* per arm	Mean difference (True value = 2)			
		Est	Emp Var	Est Var	95% Cov
		**Unadjusted**			
	50	2.01	0.327	0.342	95.3
	100	2.00	0.170	0.170	95.1
	500	2.00	0.034	0.034	95.1
		**Adjusting for *X*_1_ only**			
Covariate adjustment	50	2.00	0.107	0.110	95.7
	100	2.00	0.054	0.055	95.1
	500	2.00	0.011	0.011	95.0
IPTW	50	2.00	0.108	0.109	95.4
	(i)			0.346	99.9
	(ii)			0.108	95.3
	100	2.00	0.055	0.054	94.9
	(i)			0.171	100.0
	(ii)			0.054	95.0
	500	2.00	0.011	0.011	94.9
	(i)			0.034	100.0
	(ii)			0.011	94.9
		**Adjusting for** *X*_1_,*X*_2_ **and** *X*_3_			
Covariate adjustment	50	2.00	0.010	0.010	95.5
	100	2.00	0.005	0.005	94.7
	500	2.00	0.001	0.001	94.8
IPTW	50	2.00	0.013	0.016	96.9
	(i)			0.358	100.0
	(ii)			0.013	94.9
	100	2.00	0.006	0.006	95.1
	(i)			0.173	100.0
	(ii)			0.005	94.1
	500	2.00	0.001	0.001	94.7
	(i)			0.034	100.0
	(ii)			0.001	94.6
		**Adjusting for *X*_4_ only**			
Covariate adjustment	50	2.00	0.347	0.347	95.6
	100	1.99	0.175	0.171	94.9
	500	2.00	0.034	0.034	94.8
IPTW	50	2.00	0.347	0.336	95.1
	(i)			0.340	95.3
	(ii)			0.337	95.3
	100	1.99	0.175	0.168	94.7
	(i)			0.169	94.8
	(ii)			0.168	94.7
	500	2.00	0.034	0.034	94.8
	(i)			0.034	94.8
	(ii)			0.034	94.8

(i) = Est Var is the incorrect robust estimate (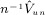); (ii) = Est Var is the ‘plug-in’ variance estimator.IPTW, inverse probability-of-treatment weighting.

Results for the analysis of binary outcomes are shown in Table 2. For covariate-adjusted estimators, the results shown are restricted to samples where convergence of the binomial outcome regression model was achieved and the estimated value was in the correct range (i.e. estimated risk differences outside the interval [-1,1] were excluded). For the estimation of the risk difference when adjusting for *X*_1_ only, 12.5%, 6.8% and 0.6% of samples encountered non-convergence of the binomial regression model for sample sizes of *n* = 50, 100 and 500, respectively, and a further 0.1% of the samples with *n* = 50 produced estimated risk differences outside the correct range. When adjusting for three baseline characteristics, 40.9%, 22.9% and 2.9% of samples encountered convergence problems in estimating the risk difference, and 0.2% and 0.1% of samples with *n* = 50 and 100, respectively, produced estimates outside the correct range. When adjusting only for an unrelated characteristic, we encountered fewer convergence problems than when adjusting for *X*_1_ only, and no estimates outside the correct range were produced. For the estimation of the risk ratio when adjusting for *X*_1_ only, 2.8%, 1.4% and 0.1% of samples encountered non-convergence of the binomial regression model for sample sizes of *n* = 50, 100 and 500, respectively. When adjusting for three baseline characteristics, 14.0%, 5.5% and 1.2% of samples encountered convergence problems in estimating the risk ratio. When adjusting only for *X*_4_, we encountered fewer convergence problems than when adjusting only for *X*_1_. In contrast, the logistic regression model converged for all samples. Similarly, we estimated all estimands using the IPTW approach for all samples, and no values fell outside the correct range.

**Table 2:** Estimated risk differences, log risk ratios and log odds ratios from *n* = 5,000 simulated datasets, with sample sizes per arm (*n*) of 50, 100 and 500. The mean treatment effect estimate (Est), the empirical variance across simulations (Emp Var), the mean of the variance estimates (Est Var) and the coverage of the 95% confidence interval (95% Cov) calculated from that variance estimate are shown.

Adjustment Method	n per arm	Risk difference (True value = 0.07)				Log risk ratio (True value = 0.31)				Log odds ratio (True value = 0.4)			
		Est	Emp	Est	95%	Est	Emp	Est	95%	Est	Emp	Est	95%
		Var	Var		Cov		Var	Var	Cov		Var	Var	Cov
		**Unadjusted**											
	50	0.07	0.007	0.007	94.8	0.33	0.169	0.169	96.8	0.42	0.333	0.263	95.8
	100	0.07	0.003	0.003	95.1	0.32	0.080	0.079	95.2	0.40	0.126	0.125	95.4
	500	0.07	0.001	0.001	94.4	0.31	0.015	0.015	95.2	0.40	0.024	0.024	94.8
		**Adjusting for *X*_1_ only**											
Covariate adjustment	50	0.06	0.007	0.006	93.3	0.32	0.172	0.168	96.2	0.43	0.341	0.270	95.7
	100	0.07	0.003	0.003	94.9	0.32	0.081	0.079	95.0	0.41	0.128	0.127	95.4
	500	0.07	0.001	0.001	94.5	0.31	0.015	0.015	95.0	0.40	0.025	0.024	94.7
IPTW	50	0.07	0.007	0.007	94.6	0.33	0.171	0.166	96.4	0.42	0.333	0.259	95.5
	(i)			0.007	94.7			0.169	96.6			0.263	95.7
	(ii)			0.007	94.3			0.168	96.2			0.300	96.7
	100	0.07	0.003	0.003	95.1	0.32	0.080	0.078	95.0	0.40	0.126	0.124	95.3
	(i)			0.003	95.1			0.079	95.2			0.125	95.4
	(ii)			0.003	95.0			0.078	95.2			0.145	96.7
	500	0.07	0.001	0.001	94.5	0.31	0.015	0.015	95.1	0.40	0.024	0.024	94.6
	(i)			0.001	94.6			0.015	95.1			0.024	94.7
	(ii)			0.001	94.4			0.015	95.0			0.028	96.4
		**Adjusting for *X*_1_,*X*_2_ and *X*_3_**											
Covariate adjustment	50	0.06	0.007	0.006	90.7	0.31	0.182	0.167	95.0	0.43	0.316	0.284	95.0
	100	0.06	0.003	0.003	94.1	0.30	0.751	0.078	95.3	0.41	0.132	0.130	95.3
	500	0.07	0.001	0.001	94.9	0.31	0.015	0.015	95.4	0.41	0.024	0.024	95.2
IPTW	50	0.07	0.007	0.007	94.0	0.32	0.182	0.167	95.2	0.42	0.293	0.257	94.4
	(i)			0.007	94.4			0.174	95.6			0.269	95.1
	(ii)			0.007	94.1			0.172	95.2			0.306	96.1
	100	0.07	0.003	0.003	94.9	0.32	0.081	0.078	95.7	0.40	0.126	0.124	95.2
	(i)			0.003	95.4			0.080	96.0			0.127	95.4
	(ii)			0.003	94.9			0.079	95.8			0.146	96.9
	500	0.07	0.001	0.001	94.9	0.31	0.015	0.015	95.3	0.40	0.024	0.024	95.3
	(i)			0.001	95.0			0.015	95.4			0.024	95.4
	(ii)			0.001	95.0			0.015	95.3			0.028	96.8
		**Adjusting for *X*_4_ only**											
Covariate adjustment	50	0.06	0.007	0.007	94.1	0.33	0.176	0.169	96.2	0.43	0.280	0.269	95.7
	100	0.07	0.003	0.003	94.4	0.31	0.081	0.079	95.2	0.41	0.128	0.126	95.2
	500	0.07	0.001	0.001	95.2	0.31	0.015	0.015	95.2	0.40	0.024	0.024	95.8
IPTW	50	0.07	0.007	0.007	95.2	0.33	0.178	0.168	96.0	0.42	0.273	0.260	95.6
	(i)			0.007	95.3			0.170	96.2			0.263	95.7
	(ii)			0.007	94.7			0.168	95.3			0.300	96.6
	100	0.07	0.003	0.003	94.7	0.32	0.081	0.079	95.2	0.40	0.127	0.124	95.1
	(i)			0.003	94.7			0.079	95.2			0.125	95.2
	(ii)			0.003	94.7			0.079	95.2			0.145	96.7
	500	0.07	0.001	0.001	95.2	0.31	0.015	0.015	95.1	0.40	0.024	0.024	95.8
	(i)			0.001	95.2			0.015	95.2			0.024	95.8
	(ii)			0.001	95.3			0.015	95.1			0.028	97.0

(i) = Est Var is the incorrect robust estimate (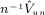); (ii) = Est Var is the ‘plug-in’ variance estimator.IPTW, inverse probability-of-treatment weighting.

For the estimation of the risk difference, covariate-adjustment using a binomial outcome regression model resulted in a slight under-estimate of the treatment effect with the smaller sample sizes (50 and 100 per treatment arm). These models also slightly under-estimated the variance, leading to slightly lower coverage probabilities. With 50 people per arm, the coverage probability of the treatment effect from a binomial regression model adjusting for the three baseline characteristics was only 0.907. The IPTW approach, conversely, showed no evidence of bias and produced coverage probabilities closer to the nominal value.

For the estimation of the risk ratio, where the binomial outcome model converged, the estimates appear to have good statistical properties. The IPTW estimators provide estimates that are similar to those from the covariate-adjustment. For the odds ratio, all methods appear to have good statistical properties. There is no evidence of bias, and most coverage probabilities were around 0.95. The one exception is for the ‘plug-in’ variance estimators for the IPTW approach. These tended to overestimate the variance, resulting in coverage probabilities that were a little larger than the nominal value.

## 7 Example

To illustrate the use of IPTW estimators in randomised trials, we analyse data taken from an individually randomised placebo-controlled trial of physiotherapy for the treatment of adhesive capsulitis (frozen shoulder or painful stiff shoulder). 27

### 7.1 Description of the data

In the original trial, 156 participants over 18 years of age with shoulder pain and stiffness of at least 3-month duration were randomised to either placebo (sham ultrasound) or physiotherapy (manual therapy and directed exercise), each given twice weekly for 4 weeks. Shoulder pain and function were assessed at baseline, 6, 12 and 26 weeks, and participant-perceived success of the intervention was assessed at 6, 12 and 26 weeks post-randomisation. The primary measure of shoulder pain was the Shoulder Pain and Disability Index (SPADI), a score ranging from zero to 100 with higher scores representing more pain/disability. The investigators assessed a range of function and active motion measures, including total shoulder flexion, which can range from 0 to 180 with higher numbers indicating a greater degree of motility. At post-randomisation follow-up visits, participants rated their recovery on a 5-point ordinal scale from 1 (worsening) to 5 (marked recovery). These were dichotomised into improvement (score of 5) versus no improvement (scores 1–4). At each follow-up time, the original analysis used a covariate-adjustment approach: linear regression models for continuous outcomes adjusting for the baseline measurement of the outcome and binomial regression models with a log-link to estimate the risk ratio for binary outcomes.

The trial concluded that physiotherapy provided no benefits for pain, function or quality of life outcomes but resulted in improvement in shoulder movement and increased participant-perceived improvement. In particular, the estimated mean difference in SPADI at 12 weeks was − 3.2 (95% CI: − 9.3, 2.9), *p* = 0.302. The estimated mean difference in total shoulder flexion at 12 weeks was 9.5 (95% CI: 2.9, 16.0), *p* = 0.005. Finally, the relative risk for improvement was 1.4 (95% CI: 1.1, 1.8), *p* = 0.016.

We have taken a random sample of approximately 80% of the participants from the trial for our current analysis. We estimate the effect of physiotherapy on the three outcome measures mentioned earlier—total shoulder flexion, SPADI and participant-perceived improvement—at 12 weeks post-randomisation. These analyses are for illustration of the methods only; clinical implications of the trial are discussed in the original paper 27.

### 7.2 Statistical analysis

We conducted all statistical analyses using Stata version 11.1 28. For the two continuous outcomes, we calculated the unadjusted difference in means between treatment groups at 12 weeks. We obtained covariate-adjusted estimates, adjusting for the baseline measurement of the outcome, from linear regression models for each outcome including the baseline outcome measurement and treatment arm as independent variables. We calculated IPTW estimates of the mean difference. For the latter, we estimated the propensity score separately for each continuous outcome, using a logistic regression model of treatment on the baseline value of the continuous outcome variable. For the IPTW treatment effect estimates, we report the usual robust standard errors from a probability-weighted regression model (using Stata′s ‘pweight’ option) of outcome on treatment, weighting by the inverse of the probability of receiving the treatment actually received. We also report corrected standard errors calculated from Equation (4).

For the binary outcome—patient-perceived improvement at 12 weeks—we calculated the unadjusted risk difference, risk ratio and odds ratio. We obtained covariate-adjusted estimates, adjusting for baseline pain and function as measured by SPADI, from a binomial regression model of outcome on treatment using the appropriate link function (via Stata′s ‘binreg’ command). We calculated IPTW estimators, using a propensity score estimated from a logistic regression model of treatment on baseline SPADI score.

### 7.3 Results

Our sub-sample of data contained 122 participants, 62 (50.8%) of whom were in the active arm with the remaining 60 (49.2%) in the placebo arm. Table 3 shows the baseline demographics of these participants. There was a moderate partial correlation of 0.50 between baseline and follow-up shoulder flexion after adjusting for treatment arm, and a partial correlation of 0.46 between SPADI measurements at baseline and 12-week follow-up. Patient-perceived improvement was negatively correlated ( correlation = − 0.19) with baseline SPADI (participants with higher pain at baseline tended to report less improvement).

**Table 3:** Demographic and clinical characteristics of the sub-sample of data.

Characteristic	Physiotherapy (*n* = 62)		Placebo (*n* = 60)	
Age (yrs), mean (SD)	55.1	(9.8)	56.0	(7.4)
Female, *n* (%)	41	(66.1)	31	(51.7)
Left shoulder affected, *n* (%)	39	(52.7)	35	(47.3)
Duration of symptoms (months), median (Q1,Q3)	6	(4, 10)	6	(4, 8)
SPADI, mean (SD)	60.2	(21.5)	60.5	(20.5)
Shoulder flexion (range 0–180), mean (SD)	92.1	(21.8)	93.7	(25.0)

SD =  standard deviation; Q1,Q3  =  25th and 75th percentiles; SPADI =  Shoulder and Pain Disability Index.

Table 4 shows the estimates of the effect of physiotherapy. For the first continuous outcome measure, total shoulder flexion, the unadjusted mean difference was 6.87 ( SE = 3.88), with 95% confidence interval of ( − 0.8,14.5) and a *p*-value of *p* = 0.08. The covariate-adjusted estimate of effect, adjusting for baseline shoulder flexion, was slightly increased to 7.62 (95% CI: 0.95,14.3), *p* = 0.03. The IPTW estimated mean difference was identical to the covariate-adjusted estimate, but the uncorrected standard error was the same as the unadjusted difference in means, resulting in a wide confidence interval and a *p*-value of *p* = 0.05. Correcting the standard error for the estimation of the propensity score gave a similar standard error to the covariate-adjusted estimate and consequently a similar confidence interval and *p*-value. For this outcome, adjustment for the baseline value resulted in a decrease in variance of approximately 25%.

**Table 4:** Estimated treatment effects for the randomised trial of shoulder pain.

Analysis	Estimate	SE	95% CI		*p*-value
**Continuous outcomes**					
*Total shoulder flexion*					
Unadjusted	6.87,	3.88	(-0.80,	14.54)	0.079
Covariate adjustment (linear regression)	7.62,	3.37	(0.95,	14.29)	0.026
IPTW (uncorrected SE)	7.62,	3.88	(0.02,	15.22)	0.049
IPTW (corrected SE)	7.62,	3.34	(1.07,	14.17)	0.023
*SPADI*					
Unadjusted	-2.05,	3.55	(-9.08,	4.98)	0.565
Covariate adjustment (linear regression)	-1.91,	3.16	(-8.16,	4.35)	0.547
IPTW (uncorrected SE)	-1.91,	3.56	(-8.88,	5.06)	0.592
IPTW (corrected SE)	-1.91,	3.14	(-8.06,	4.24)	0.544
**Binary outcome**					
*Improvement (logarithm of risk ratio)*					
Unadjusted	0.30,	0.139	(0.03,	0.57)	0.031
Covariate adjustment (binomial regression)	Convergence not achieved				
IPTW (uncorrected SE)	0.30,	0.139	(0.02,	0.57)	0.033
IPTW (corrected SE)	0.30,	0.137	(0.03,	0.57)	0.030
*Improvement (risk difference)*					
Unadjusted	0.19,	0.085	(0.03,	0.36)	0.024
Covariate adjustment (binomial regression)	0.20,	0.080	(0.04,	0.35)	0.015
IPTW (uncorrected SE)	0.19,	0.085	(0.02,	0.36)	0.026
IPTW (corrected SE)	0.19,	0.084	(0.03,	0.35)	0.023
*Improvement (logarithm of odds ratio)*					
Unadjusted	0.86,	0.389	(0.09,	1.62)	0.028
Covariate adjustment (binomial regression)	0.89,	0.400	(0.11,	1.66)	0.026
IPTW (uncorrected SE)	0.85,	0.391	(0.08,	1.61)	0.030
IPTW (corrected SE)	0.85,	0.383	(0.10,	1.60)	0.027

For continuous outcomes, adjusted estimates are adjusted for the baseline value of the outcome measure

For binary outcomes adjusted estimates are adjusted for baseline SPADI.

IPTW =  inverse probability-of-treatment weighting; SPADI =  Shoulder and Pain Disability Index.

For the second continuous outcome, SPADI, we see a similar pattern. All estimates of treatment effect were in the direction of improved outcomes in the physiotherapy arm, but none reach statistical significance. Adjustment for the baseline value of the outcome resulted in approximately a 20% reduction in variance. For the IPTW estimate, this reduction was only seen when the corrected standard error was calculated.

We quantified the treatment effect for the binary outcome, patient-perceived improvement, using the risk ratio (the estimand in the original trial) and also using the risk difference and odds ratio, for illustration. The unadjusted log risk ratio was 0.3 (95% CI: 0.03,0.57), equivalent to a risk ratio of 1.35 (95% CI: 1.03,1.77), with *p* = 0.03. Participants in the physiotherapy arm had an increased chance of perceiving an improvement at 12-week post-randomisation. Calculating a covariate-adjusted risk ratio, adjusting for the baseline SPADI, was not possible; convergence was not achieved. Conversely, we calculated adjusted treatment effect estimates using IPTW. The point estimate was identical to the unadjusted estimate with a slightly decreased standard error, although the correct formula for the standard error was required to achieve this variance reduction. The magnitude of the variance reduction achieved by adjustment (approximately 2%) was much smaller than that seen for continuous outcomes.

When estimating the risk difference, we achieved adjustment for baseline SPADI using both a binomial regression model adjusting for SPADI and via IPTW. In this case, we see that variance reduction achieved by covariate-adjustment was bigger than the reduction achieved using IPTW. However, we saw in the simulation study that the variance of the estimated risk difference was often slightly underestimated by the outcome regression model with small sample sizes like this one. So the larger variance reduction achieved by the outcome regression model may not reflect a real increase in precision.

Logistic regression adjusting for baseline SPADI yielded, as expected, a conditional adjusted log odds ratio further away from the null value of zero, a change from 0.86 to 0.89. At the same time, the standard error was slightly increased, but the overall effect of the adjustment took the *p*-value from *p* = 0.028 (unadjusted) to *p* = 0.026 (covariate-adjusted using logistic regression). In contrast, the adjusted IPTW estimate was very similar to the unadjusted estimate, but the standard error was slightly decreased.

## 8 Discussion

We have demonstrated that propensity scores are a useful tool for analysing randomised studies where pre-specified adjustment for a baseline characteristic is planned. Although modelling the propensity score is often viewed as modelling the treatment allocation process, the process can be viewed as an adjustment for chance imbalance of prognostic variables. Particularly in randomised trials, predictors of outcome can, and should, be included in the (pre-specified) propensity score model in order to account for chance imbalances. In a randomised setting, the propensity score IPTW analysis can be pre-specified in the same way as is performed for other common covariate-adjustment approaches, thereby avoiding biases due to post-hoc modelling decisions.

For continuous outcomes, we have shown that the IPTW estimator has large-sample statistical properties similar to covariate-adjustment via a linear regression model, in scenarios where the linear regression modelling assumptions hold. In our shoulder-pain example, where the linear regression model assumptions appeared to hold, for our moderately-correlated baseline and follow-up measurements of continuous variables, adjustment for the baseline value resulted in between 20% and 25% reduction in variance of the treatment effect estimate using either linear regression or the IPTW estimator. This variance reduction would be greater for variables with higher correlation. However, using an IPTW estimate with the uncorrected standard error could lead to incorrect inference and conclusions being drawn.

In scenarios where the linear regression model does not hold, for example, where the assumption of a linear relationship between the baseline and outcome measurements is false, the IPTW estimator may be preferable. We are planning future work in which we will examine the relative merits of covariate-adjustment and IPTW in cases where the linear regression model is misspecified.

For binary outcomes, particularly rare binary outcomes, covariate-adjustment on a risk difference or risk ratio scale may not be possible because of instability of these models. Adjustment using the propensity score approach will, conversely, be possible. This is illustrated in our shoulder-pain example and in our previous work on risk differences in observational studies 29. When analysing the binary outcome of patient perceived improvement, we were able to obtain covariate-adjustment estimates of the risk difference but not the risk ratio. In contrast, adjustment using IPTW was successful in providing an estimate for all three estimands. Convergence will always be achieved with the IPTW estimators because they are simply based on weighted sample means.

When the odds ratio is of interest but adjustment is necessary, the IPTW approach has the advantage of preserving the original estimand, the marginal odds ratio, whereas covariate-adjustment via logistic regression changes the target parameter because of non-collapsibility of the odds ratio. However, further work is required to determine which of these approaches provides the greatest statistical power to detect a treatment effect.

We have only considered one propensity score method—inverse probability of treatment weighting. Other methods that use the propensity score, such as matching or sub-classification on the propensity score or including the estimated propensity score in an outcome regression model, may be less useful in this context. In particular, matching would have the undesirable feature of omitting some randomised individuals from the analysis. Further, all these methods estimate a conditional estimand, in contrast to the marginal IPTW estimand; thus, care is required when estimating odds ratios. However, this is the same for the commonly used adjusted logistic regression models so is not necessarily a barrier to the use of these methods. Further research evaluating the use of other propensity score methods in randomised trials is needed to establish the utility of these methods in this context.

We have also considered only a logistic parameterization for the propensity score model. Other approaches, for example, neural networks or boosting, may perform better. We would require further research to identify situations in which such models provided further variance reductions in comparison with the logistic regression model.

We have considered large-sample results in this paper. Further work will be needed to investigate small sample properties of these estimators. On the basis of our limited work to date, we expect these results to hold for fairly small sample sizes, for example 50 participants per arm.

In summary, we can perform pre-planned adjustment for baseline characteristics in individually randomised trials using inverse probability weighted estimators based on the estimated propensity score. These estimators have comparable statistical properties to more common alternatives and, in certain circumstances, may be a preferable analysis method. The propensity score approach would increase the amount of pre-specification, by allowing the adjustment to be undertaken prior to outcome data collection, thus removing any possibility of bias due to influence of the outcome data on the chosen analysis approach.

## Appendix

### Appendix

The unknown parameter is ***θ*** = (*μ*_1_,*μ*_0_,***α***^ ⊤ ^)^ ⊤ ^, with the first two components as scalars and the third a *p* × 1 vector. Partitioning the matrix **B** as for ***θ*** and noticing that , we have

Similarly, because *∂e* / *∂****α***^ ⊤ ^ = **x**^ ⊤ ^*e*(1 − *e*) and so *∂e*^ − 1^ / *∂****α***^ ⊤ ^ = − **x**^ ⊤ ^*e*^ − 1^(1 − *e*) and *∂*(1 − *e*)^ − 1^ / *∂****α***^ ⊤ ^ = **x**^ ⊤ ^*e*(1 − *e*)^ − 1^,

**Large sample variance:** By conditioning on **x** and using the equality  and replacing the observed by the potential outcomes, we can simplify these expressions, giving , , , , and *b*_33_ = *a*_33_. Also, we have *a*_11_ = *a*_22_ = 1.

After simplifying **A** and **B** using these large-sample equalities, direct matrix multiplication gives

The multivariate delta method states that for a function *f*(**w**) of two random variables, **w** = (*w*_1_,*w*_2_)^ ⊤ ^, the large-sample variance of *f* is given by *∂f* / *∂***w**^ ⊤ ^*Cov*(**w**) *∂f* / *∂***w**. Applying this to , for *j* = 1,2,3, gives

where *K*_11_ = *K*_10_ = 1, ,  and . Substituting in expressions for the submatrices of **A** and **B** gives the expression in the main text, Equation (2).

**Sample estimate of variance:** Rather than simplifying the expectations in **A** and **B**, we simply estimate each component by replacing the expectation by a sample average. Using hats to denote sample estimates,

Applying the delta method gives the variance in the text, Equation (4), noting that we have let ,  and .

**Omitting estimation of propensity score:** If the propensity score is a known, or specified function, we repeat the aforementioned calculations omitting the third row of **u**, (**x**(*Z* − *e*)), which results in the deletion of the third columns and rows of matrices **A** and **B**.
